# Optical Properties of Au-Based and Pt-Based Alloys for Infrared Device Applications: A Combined First Principle and Electromagnetic Simulation Study

**DOI:** 10.3390/mi10010073

**Published:** 2019-01-20

**Authors:** Min-Hsueh Chiu, Jia-Han Li, Tadaaki Nagao

**Affiliations:** 1 Department of Engineering Science and Ocean Engineering, National Taiwan University, No. 1, Sec. 4, Roosevelt Rd., Taipei 10607, Taiwan; peter810601@gmail.com; 2 International Center for Materials Nanoarchitectonics (WPI-MANA), National Institute for Materials Science (NIMS), 1-1 Namiki, Tsukuba, Ibaraki 305-0044, Japan; 3 Department of Condensed Matter Physics Graduate School of Science, Hokkaido University, Kita-10 Nishi-8 Kita-ku, Sapporo 060-0810, Japan

**Keywords:** alloy, infrared device, gold, platinum, permittivity, quality factor, first principle

## Abstract

Due to the rapid progress in MEMS-based infrared emitters and sensors, strong demand exists for suitable plasmonic materials for such microdevices. We examine the possibility of achieving this goal by alloying other metals with the noble metals Au and Pt, which have some drawbacks, such as low melting point, structural instability, and high costs. The six different metals (Ir, Mo, Ni, Pb, Ta, and W) which possess good properties for heat resistance, stability, and magnetism are mixed with noble metals to improve the properties. The optical properties are calculated by density functional theory and they are used for further investigations of the optical responses of alloy nanorods. The results show that the studied alloy nanorods have wavelength selective properties and can be useful for infrared devices and systems.

## 1. Introduction

According to Planck’s law, the thermal radiation of an ideal black body depends only on its temperature. However, in general, the thermal emissions from realistic objects differ substantially depending on their dielectric response and surface morphologies. Theoretical study and engineering designs of thermal emission devices are not only important in thermal emitters and infrared sensors but also useful for MEMS-based systems and micromachines. Therefore, some researchers are aiming to artificially tailor the emission spectrum by selecting the constituent materials and rationally designing their surface nanostructures to utilize thermal energy and heat management in a more efficient manner. For example, Dao et al. [1] demonstrated mid-infrared metamaterial perfect absorbers by using Al-Al_2_O_3_-Al tri-layers structure, which an exhibited excellent absorptivity of 98% and a flexible wavelength selective response in the infrared region tuned by the disk diameter. They also demonstrated that the sharpness of the emission peak of the device, which only uses inexpensive industrial material, was comparable to that of absorbers composed of gold or silver. Yang et al. [2] applied four elemental metals (Al, Au, Mo, W) to realize a Tamm plasmon polariton to achieve a narrow band thermal emission, which may efficiently confine the energy and result in a sharp peak in absorption spectrum in the infrared region. Yokoyama et al. [3] achieved high emission intensity by developing a thermal emitter operative at very high temperature of 1000 °C using refractive molybdenum and aluminum oxide in a metal–insulator–metal structure. Both reported the wavelength selective characteristics by adequately selecting the material and structural parameter, to achieve higher device performance in the infrared region. Regarding the operation temperature, it is an important factor which may cause the degradation of material and damage to the devices. Therefore, exploring new refractory materials is expected to bring about greater potential to work in high-temperature environments. TiN thin film has suitable plasmonic properties similar to Au as well as a substantially high thermal stability up to about 500 °C [4]. Kaur et al. [5] combined TiN nanoparticles and transparent ceramic microfiber wools to efficiently desalinate water by the broadband photothermal heat generation by absorbing ultraviolet to near-infrared light. Sugavaneshwar et al. [6] demonstrated high-quality TiN film on a flexible polymer film at room temperature, and illustrated the better and swifter conversion from light to heat energy in the infrared region.

On the other hand, due to the strong transmission and low hazard of infrared light to the living body, it is used for non-destructive sensing applications. For example, surface-enhanced Raman spectroscopy (SERS) is a technique widely used detection and sensing microdevices. With localized surface plasmons, Bansal et al. [7] simulated the effects of scattering efficiencies on the different shapes of Ag-Cu alloy, such as sphere, cube, and nanobar with an effective radius 50 nm. Dodson et al. [8] adjusted the gap between bowties to be 10 nm to pursue an extremely high enhancement factor. The effects of amplifying the electric field can be utilized in a sparse solution. The object in sparse solution can be concentrated with magnetic nanoparticles by applying the magnetic field, which reinforces the sensing efficiency. Brullot et al. [9] simulated and discussed the relations between the structure, size, and optical properties of magnetite-core gold-shell nanoparticles. They developed the comprehensive mechanism for designing the core-shell nanoparticles in the near-infrared light region. Kwizera et al. [10] successfully synthesized different shapes (sphere, popcorn, and star) and sizes of iron-oxide-core and gold-shell nanoparticles. The optical and magnetic properties of nanoparticles that may be exploited in different applications have been demonstrated. Liu et al. [11] synthesized urchin-like Ag-coated Ni nanoparticles. The rhodamine-6G sparse solution can be easily detected due to the large amount of hot spots. It has also been shown that such nanoparticles are quickly and simply manipulated by a magnetic field due to their nickel cores. Zhang et al. [12] applied nanoparticles in realistic applications and showed the differentiation of three different bacteria using Fe_3_O_4_-Au core-shell nanoparticles, and the signal was about 60 times higher than that obtained without a magnetic field.

Most bio-molecules vibrate in the infrared light, hence, infrared light is widely used in bio-sensing applications. Also, plasmonic nanoparticles surrounded by a catalyst are believed to improve the activities for the chemical transformations, e.g., by decreasing the chemical potential and increasing the number of free electrons. Both procedures increase the efficiency of decomposition reactions. Song et al. [13] provided an exhaustive investigation of the mechanism by which the photocatalytic reduction of carbon dioxide is achieved. The results revealed that the CO_2_ decomposition efficiency of Au-Pt-SiO_2_ nanoparticles was about twice as high compared to Pt-SiO_2_ nanoparticles under the light intensity of 0.6 (W/cm^2^); the efficiency of Au-Pt-SiO_2_ nanoparticles was found to be better than that of Au-SiO_2_ and Pt-SiO_2_ nanoparticles. Bora et al. [14] discussed the surface temperature of Au-ZnO nanorods under resonant excitation up to about 300 °C, which gave an apparent quantum yield (for the degradation of methylene blue) about 6 times higher than that achieved by bare ZnO nanorods. Mukherjee et al. [15] showed that the H_2_ dissociation can be achieved at room temperature due to the surface plasmon-induced hot electrons at the Au nanoparticle surface, as H_2_ has an extremely high dissociation energy (4.51 eV).

The operation frequency is one of the most important factors influencing device efficiency. Some researchers have tuned the structure parameters of nanoparticles, as discussed above, to achieve the desired result. Instead of changing the morphology of the microstructures or nanostructures, one could focus on the optical properties of the selected materials, which are key parameters that strongly affect the plasmonic responses of materials. Different classes of materials have been explored, and they were found to depend on the operating wavelength of the applications. Some materials give a relatively low imaginary part of the permittivity as compared to metallic layers, such as graphene [16] or indium tin oxide [17,18]. Although graphene has been employed as a plasmonic material and has a negative real part of the permittivity under suitable operating conditions in the infrared light region, its magnitude of the real part of the permittivity is not so large and it gives a lower electric field enhancement on the surface of materials. To mitigate the loss of metallic materials (low imaginary part of permittivity) and maintain a relatively strong electric field enhancement (high magnitude of real part of permittivity), we propose a method of adding other components to obtain a hybrid alloy. The operating wavelength of a material can be adjusted with different atomic ratios for different applications. Silva et al. [19] gave the experimental and analytic data of AuCu alloy, which illustrated that the AuCu alloy possesses superior plasmonic properties from 500 nm to 690 nm. Keast et al. [20] simulated a series of Au-based intermetallic materials (Al, Cd, Mg, Pd, Pt, Sn, Zn, Zr) and discussed the localized surface plasmon properties of the materials. Gong et al. [21] synthesized alloys combining two out of Au, Ag, and Cu, which give a tunable wavelength characteristic in the visible light region. The authors also mentioned that the permittivity of the alloys cannot be approximated by a simple linear combination of the dielectric function from the different metals making up the composition of the material. However, most of the strong plasmonic effects of noble metals occur in the visible region, which is dominated by interband transition. Blaber et al. [22] also stated that the partially occupied d bands of the alloy may affect the efficiency of its plasmonic effects, but the dopants may disrupt the low energy transition, which would in turn increase the plasmonic quality. Hence, the alloy may suitable for applications operating in the infrared region.

In this work, we explore the materials that will be potentially operated in the infrared region, and discuss Au-based and Pt-based noble metal alloys mixed with other six metals (Ir, Mo, Ni, Pb, Ta, W). Au and Pt are widely used in the fields of plasmonics and catalysis. The permittivity of the noble metals supports a very good quality surface plasmon in the visible to infrared regions, and they are stable in normal environments. However, Au has a relatively low melting point (1377 K), while Pt has a slightly higher melting point (2041 K) but a rather high loss compared to Au. It is predicted that the melting point will increase when the alloy is combined with refractory metals such as Ir, Mo, Ta, and W, whose melting points are 2739, 2896, 3290, and 3698 K, respectively. It is shown that the tensile strength of the alloys increases with the inclusion of Ir in PtIr [23]. Moreover, Ni-based alloys may also exhibit magnetic properties due to their ferromagnetic characteristic, and thus they will be useful in nano-bio applications. It is also predicted that the thermal stability of alloys will increase with the introduction of Ni. In addition to the transition metal, the poor metal is also discussed. Compared to the transition metals, the poor metals have a higher electronegativity, a lower melting point, and may be used in organic and biomolecule applications. Pb is one of the poor metals that is reported to show plasmonic properties in nanoparticles in the ultraviolent region [24]. In this work, we focus on the infrared optical properties of these unexplored alloys and discuss their potential to be used in infrared devices and systems. To investigate the optical responses of some practical nano objects, nanorods made of pure metals and different alloy materials are examined by finite-different time-domain simulations. These nanorods can be applied in high-temperature environments, localized surface plasmon polaritons, and catalysts. It should be noted that some of the alloys were found to have better plasmonic properties or magnetic properties than pure elemental metals, indicating the effectiveness of the alloying strategy for exploring high-performance infrared devices.

## 2. Materials and Methods

### 2.1. Materials

In this paper, we consider bi-metal alloys which are a combination of one noble metal and another metallic element. For the studied bi-metal alloys, the noble metal is Au or Pt and the other metallic element is Ir, Mo, Ni, Pb, Ta, or W in our simulations. The simulated materials include 12 types of bi-metal alloys. The structures of the pure metallic materials in normal conditions are listed in Table 1.

**Figure 1:**
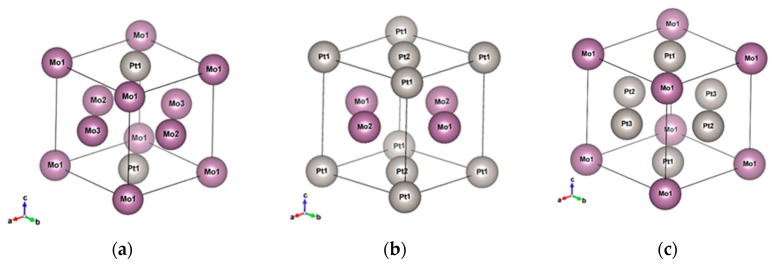
Snapshots of the Pt_x_Mo_(1−x)_ F3m3 structures with different morphologies: (**a**) Pt_0.25_Mo_0.75_, (**b**) Pt_0.5_Mo_0.5__flat, and (**c**) Pt_0.75_Mo_0.25_.

**Figure 2:**
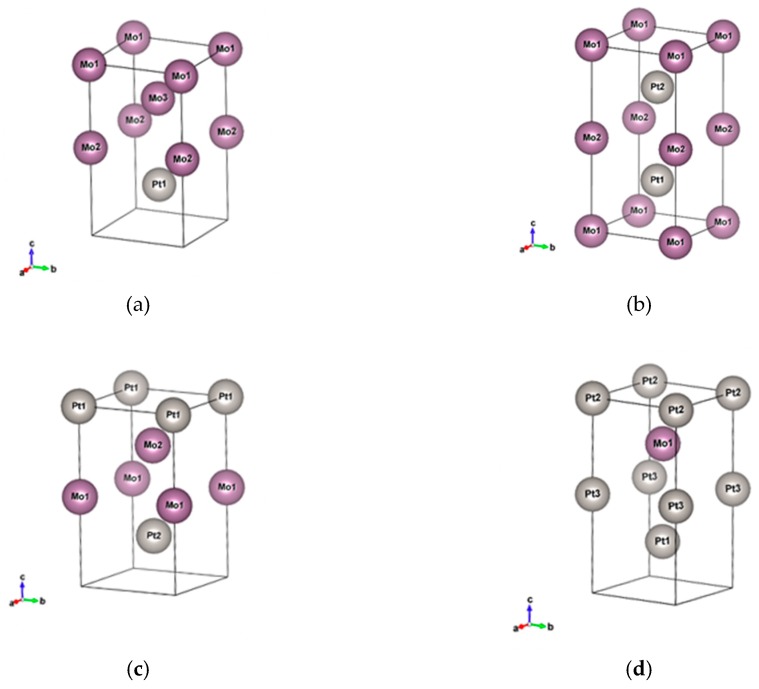
Snapshots of the Pt_x_Mo_(1−x)_ I3m3 structures with different morphologies: (**a**) Pt_0.25_Mo_0.75_, (**b**) Pt_0.5_Mo_0.5__mix, (**c**) Pt_0.5_Mo_0.5__upper, and (**d**) Pt_0.75_Mo_0.25_.

The crystal structure and electronic structure of the alloys are important factors influencing the alloys’ material properties. Theoretically, it is predicted that the crystal structure of a bi-metal alloy remains the same as its constituted elements if the constituted elements have the same crystal structure. Boschow et al. [25] showed that Au_92_Ni_8_ exhibits the same space group, F3m3, as the constituent elements. However, some researchers have showed that the crystal structure can be reshaped and transformed into other crystal structure with an equal ratio of compositions and very different Bohr radii of elements. For example, Leroux [26] recorded the phase diagram of PtNi. It was shown that the crystal structure of PtNi alloy with an atomic ratio of around 0.5 is quite different from the crystal structures of Pt-rich or Ni-rich alloys. More complicated phase diagrams of bi-metal alloys can be found for constituted elements with two different structures. Ocken et al. [27] illustrated the phase diagram of PtMo from body-centered cubic (BCC) Mo to face-centered cubic (FCC) Pt. From the Inorganic Crystal Structure Database (ICSD [28]), it was shown that that AuTa alloy is of the F3m3 space group for Au_0.9_Ta_0.1_ but has a CsCl structure for Au_0.5_Ta_0.5_. For systematic discussions, we simulated the selected 12 alloys with I3m3 and F3m3 structures which may appear in nature. The snapshots of the Pt_x_Mo_(1−x)_ F3m3 and I3m3 structures with different morphologies are shown in Figure 1 and Figure 2, respectively.

### 2.2. Methods

The results of the electronic structures of alloys were calculated using a plane wave basis with a cut-off energy of 450 eV and the generalized gradient approximation (GGA) of the Perdew–Burke–Ernzerhof (PBE) [29] exchange correlation functional. The total energy was minimized by the quasi-Newton algorithm with a limitation of 0.0001 eV/Å. Spin polarization was considered for nickel-containing alloys. The ground state was calculated with 10×10×10 k-points in momentum space. For optical permittivity calculation, a k-points grid of 50×50×50 was chosen to ensure sufficient description for Brillouin zone. All k- points divisions are according to the Monkhorst–Pack method [30].

The finite-difference time-domain (FDTD, Lumericlal) calculations were performed to predict the optical responses of the bi-metal alloy nanorods. The wavelength-dependent dielectric response of alloys was calculated using density functional theory (DFT), and then used to evaluate the optical responses of a single nanorod. The light source was set as the plane wave, which has a polarization parallel to the nanorod. The mesh size was set as one order of magnitude smaller than the structure size.

## 3. Pure Au

The permittivity of bulk gold was first calculated and compared with the experimental data [31,32,33,34,35,36,37,38,39] to check the accuracy of the computation procedures. The permittivity was constructed by the interband and intraband contributions. The intraband is related to the behavior of the free electrons, which can be depicted by Drude’s model. The real and imaginary parts of the intraband are expressed by Equations (1) and (2):

(1) Re[εintra(ω)]=1−ωp2ω2+Γ2

(2) Im[εintra(ω)]=Γωp2ω3+ωΓ2

where $\omega_{p}$ represents the intraband free electron plasma frequency and Γ is the damping frequency. In this paper, $\omega_{p}$ is calculated by DFT and Γ is obtained from the experimental data or high accuracy simulation.

**Figure 3:**
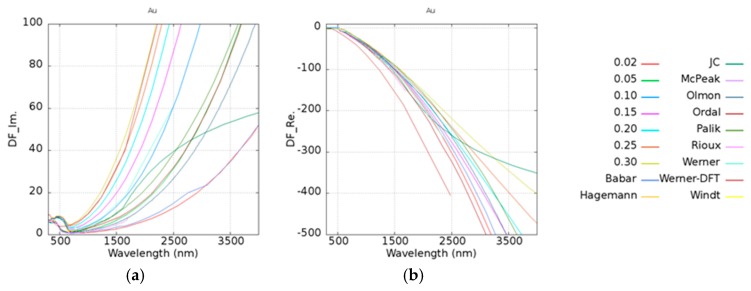
Testing of the damping frequency which varies from 0.02 to 0.3 in Au on (**a**) imaginary and (**b**) real parts of the permittivity. Different references [31,32,33,34,35,36,37,38,39] are plotted for comparison. DF_Im. and DF_Re. along the *y*-axes denote as the imaginary and real parts of the dielectric functions.

To determine the effect of Γ, we took Au as an example. In Table 2, the damping frequency is shown to fit Drude’s model according to Blaber [40], Ordal [41], and Zeman [42]. For the comparisons, the damping frequency was calculated from 0.02 to 0.3 eV, and the experimental data of imaginary and real parts of dielectric constants are shown in Figure 3a,b. DF_Im. and DF_Re. along the *y*-axes in Figure 3 are denoted as the imaginary and real parts of the dielectric functions. It was found that the trend of Badar’s data [31] which has a fitting parameter Γ with a value of 0.0184 eV, is similar to the calculated case of Γ equal to 0.02 eV. Zeman’s [42] data was fitted by Hagemann’s [32] experimental results which are located between 0.05 and 0.1 eV. Although Hagemann only provided experimental data under 826 nm, the trend of this data is similar to that of our simulation data. Olmon et al. [35] discussed the relationship between the parameter Γ and the permittivity of the different morphologies of gold in detail.

To excite the plasmons, the real part of the dielectric function should be negative to match the condition of the adjacent dielectric material. The parameter quality factor (*Q*) is introduced to examine the performance of plasmons, and it is defined as Equation (3).

(3) Q=−Re[ε(ω)]Im[ε(ω)]

**Figure 4:**
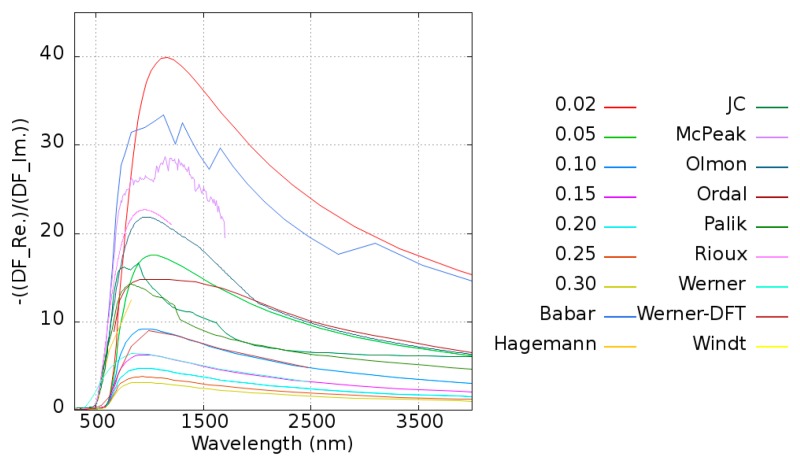
Quality factor versus wavelength for the damping frequency varying from 0.02 to 0.3 eV for Au. The quality factors calculated from the dielectric constants obtained from different references [31,32,33,34,35,36,37,38,39] are plotted for comparison.

For a given material, a high *Q* means that it has less loss and a stronger resonance property for inducing plasmons in this frequency range. The *Q* of pure Au is shown in Figure 4. The maximum of *Q* is located at around 1100 nm, which is consistent with the experimental data [31,32,33,34,35,36,37,38,39]. Most of the experimental data’s Γ are located between 0.02 and 0.1 eV. Considering the different metals employed in this work, the damping frequency is taken as 0.1 eV for all systems except pure Au.

## 4. Au-Based Alloy

The permittivity of alloys was calculated by combining the DFT calculated interband transition and Drude’s term of intraband transition. To justify the calculated results, we took AuNi as an example. Jain et al. [43] reported an AuNi alloy wire generated by melting 99.99% pure nickel and gold ingot. It was predicted that the alloy atomic ratio would be 0.5 due to the vacuum system and repeated re-melting process. The quality factors of the AuNi from Jain’s results were 0.575, 0.660, and 0.606 at 1000, 1500, and 2000 nm wavelengths, respectively. The trend of the quality factor was consistent with our data on Au_0.5_Ni_0.5_ (BCC_mix), which exhibited quality factors of 0.668, 0.686, and 0.682 at the same wavelengths.

**Figure 5:**
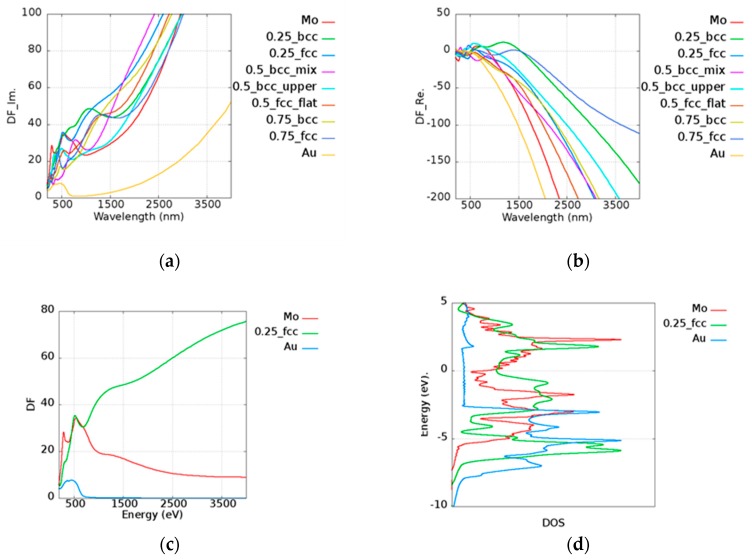
(**a**) Imaginary and (**b**) real parts of the permittivity of AuMo alloy. (**c**) The interband transition of the imaginary part of the permittivity and (**d**) the density of states of Mo, Au_0.25_Mo_0.75__FCC, and Au. DF_Im. and DF_Re. along the *y*-axes denote the imaginary and real parts of the dielectric functions.

To inspect the optical properties of alloys, we took Au_x_Mo_(1−x)_ alloy as an example. The imaginary and real parts of the permittivity of Au_x_Mo_(1−x)_ alloy are shown in Figure 5a,b, respectively. In the legend of the plots in Figure 5, the first and the last ones characterize the pure metallic elements. The number represents the atomic ratio of Au, while the text after the number denotes the structure and the morphology under the given atomic ratio condition. For example, 0.25_bcc represents Au_0.25_Mo_0.75_ with a BCC structure. In the Au_x_Mo_(1−x)_ alloy, all cases possessed a higher imaginary part of the permittivity than pure Au and Mo above 1000 nm. In addition to intraband transition, the interband transition also has the contribution to the imaginary part of the permittivity. Here, Au_0.25_Mo_0.75_ (FCC) was taken as an example for further discussion. The permittivity contributed by the interband transition of Au_0.25_Mo_0.75_ (FCC), pure Au, and Mo is shown in Figure 5c, and the corresponding density of states (DOS) is shown in Figure 5d. The DOS for each case is normalized by the maximum value within the range of −10 to 5 eV. It was shown that the onset of interband transition is roughly consistent with the distance between the large quantity states and the Fermi level. For instance, it was shown that the first huge DOS peak below the Fermi’s level is around 2.5 eV in the case of Au (Figure 5d, blue line), whereas the occurrence of interband transition is around 516 nm. The combination of Au and Mo leads to additional states, which are known as the virtual states [20] and add the possibility of photon absorptions for the higher imaginary part of the permittivity.

**Figure 6:**
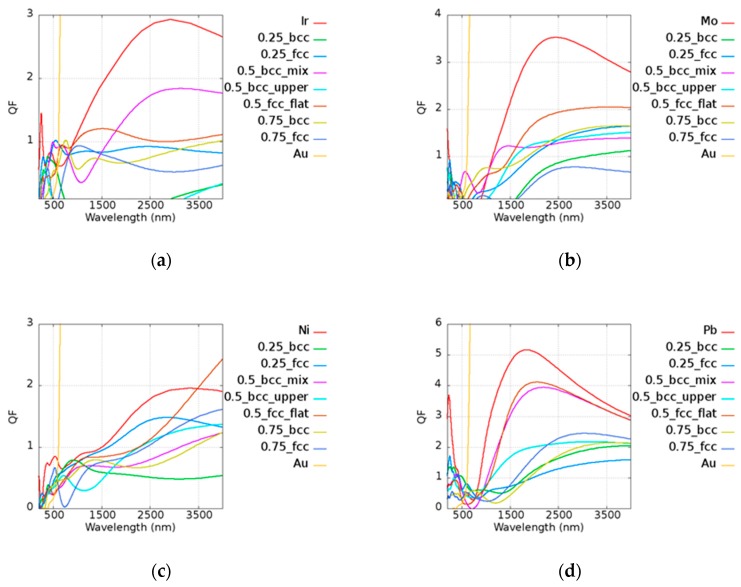

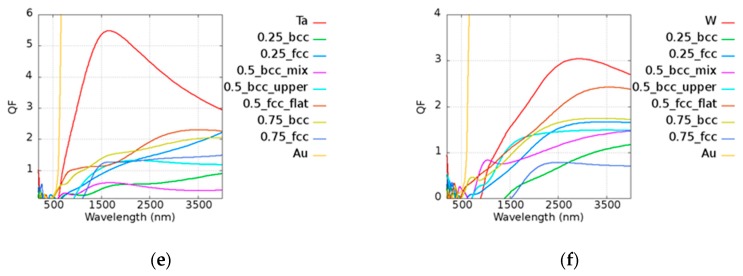
Quality factors of (**a**) Au_x_Ir_(1−x)_, (**b**) Au_x_Mo_(1−x)_, (**c**) Au_x_Ni_(1−x)_, (**d**) Au_x_Pb_(1−x)_, (**e**) Au_x_Ta_(1−x)_, and (**f**) Au_x_W_(1−x)_ alloys.

**Figure A1:**
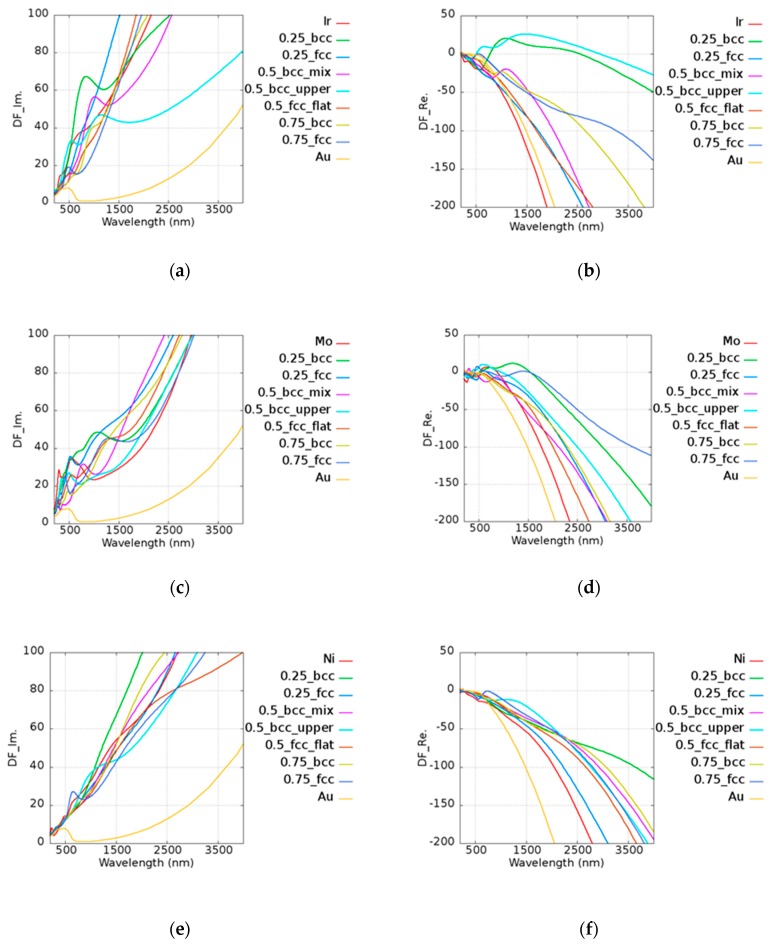

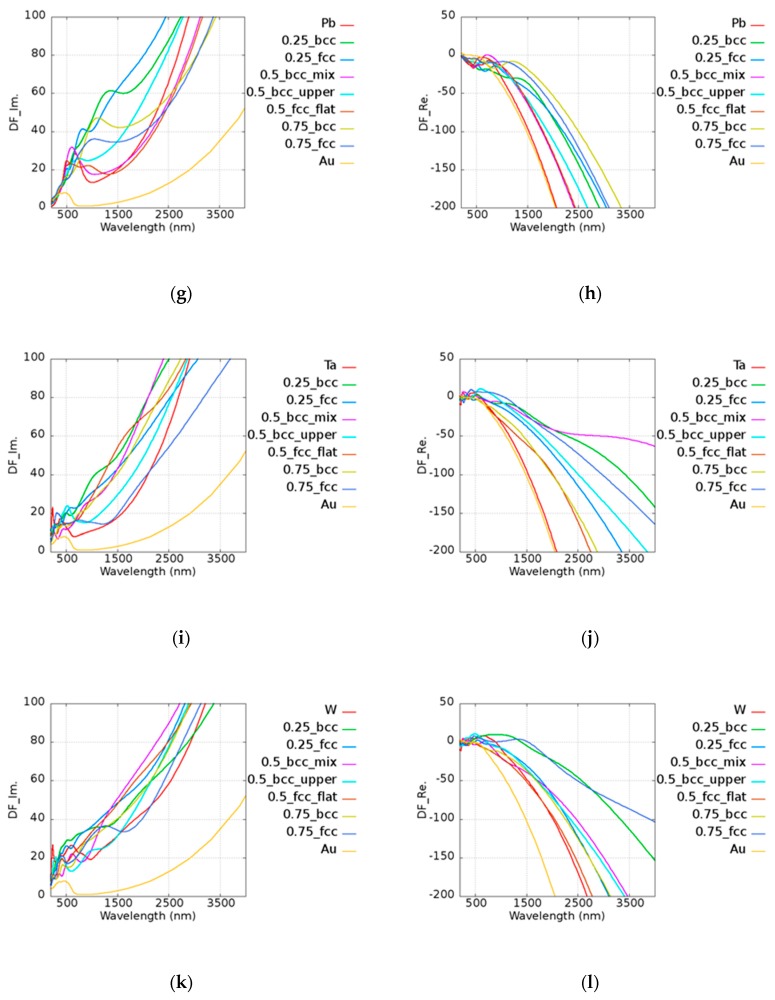
Imaginary and real parts of the permittivity of (**a**,**b**) AuIr, (**c**,**d**) AuMo, (**e**,**f**) AuNi, (**g**,**h**) AuPb, (**i**,**j**) AuTa, and (**k**,**l**) AuW. DF_Im. and DF_Re. along the *y*-axes denote the imaginary and real parts of the dielectric functions.

The results of *Q* of the Au-based alloy are shown in Figure 6. In the legend of the plots, the first and the last ones characterize the pure metallic elements. The number represents the atomic ratio ofe Au, while the text after the number denotes the structure and the morphology under the given atomic ratio condition. For example, in Figure 6a, 0.5_bcc_mix represents Au_0.5_Ir_0.5__mix with a BCC structure. The extreme high *Q* of Au is not fully shown here, although it is given in Figure 4. A material which has a *Q* value lower than 1 may not be a good plasmonic material in such a frequency. Low-*Q* materials occur around the visible region in most cases, and this contributes to the relatively high real part of the permittivity and the onset of interband transition. Some cases even serve as a dielectric material; in other words, they have a positive real part of the permittivity, as shown in the Appendix A. Although some alloys possess lower imaginary parts compared to pure metal, such as AuIr and AuNi, it was found that alloys have a larger real part of the permittivity due to the reduction of the plasma frequency. This leads to a larger intraband transition, as shown Equation (1), causing the shrinking of *Q*. Most of the cases exhibited a smaller *Q* than pure metal, but there are still several instances that showed a good property of *Q*. For example, Au_0.25_Ir_0.75_ and Au_0.75_Ir_0.25_ had a better performance than pure metal at 600 nm and 800 nm of narrow band. The extremely low imaginary part of the permittivity of Au_0.5_Ni_0.5_ (FCC) was found even compared with other Au-based alloys and pure Ni. The real and imaginary parts of permittivities of Au-based alloy are shown in Figure A1 of Appendix A. It is not surprising that Au_0.5_Ni_0.5_ (FCC) showed a good performance above 3500–4000 nm. Au_0.5_W_0.5_ (BCC_mix) possessed a better *Q* performance than pure W around the near-infrared region at 1000 nm. On the other hand, manufacturing costs may be reduced by using different compositions. The prices of Au, Pt, and Ir remained in same order in 2018, being more expensive than Ni, Mo, Pb, Ta, and W. The raw material cost may be an important issue affecting the mass production of devices. The *Q* performance was larger than 1 in most of the Au-based alloys in the infrared region, indicating that these may be used in different applications.

## 5. Pt-Based Alloy

**Figure 7:**
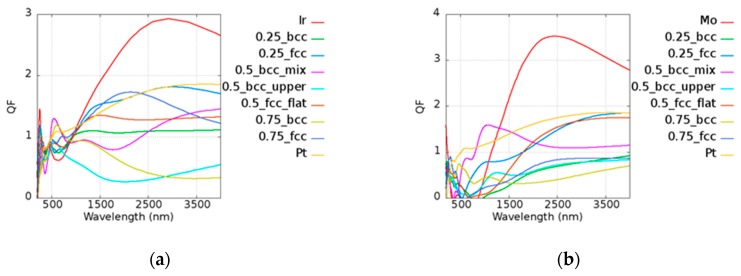

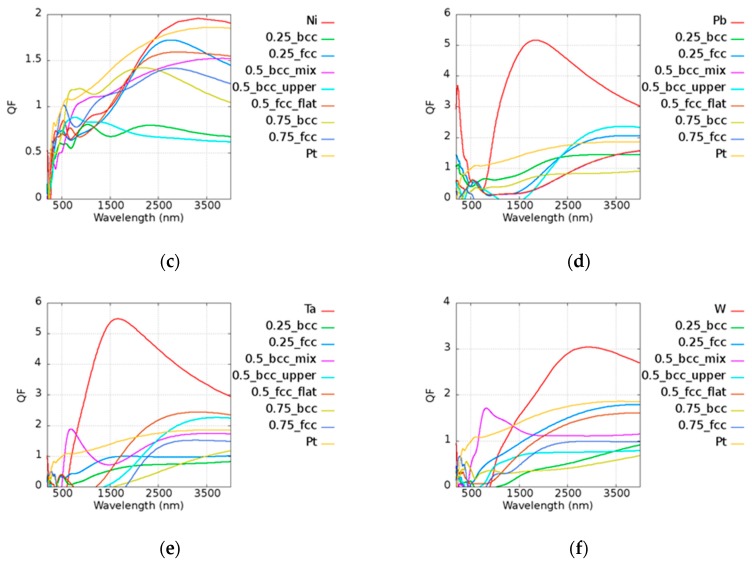
Quality factors of (**a**) Pt_x_Ir_(1−x)_, (**b**) Pt_x_Mo_(1−x)_, (**c**) Pt_x_Ni_(1−x)_, (**d**) Pt_x_Pb_(1−x)_, (**e**) Pt_x_Ta_(1−x)_, and (**f**) Pt_x_W_(1−x)_ alloys.

**Figure A2:**
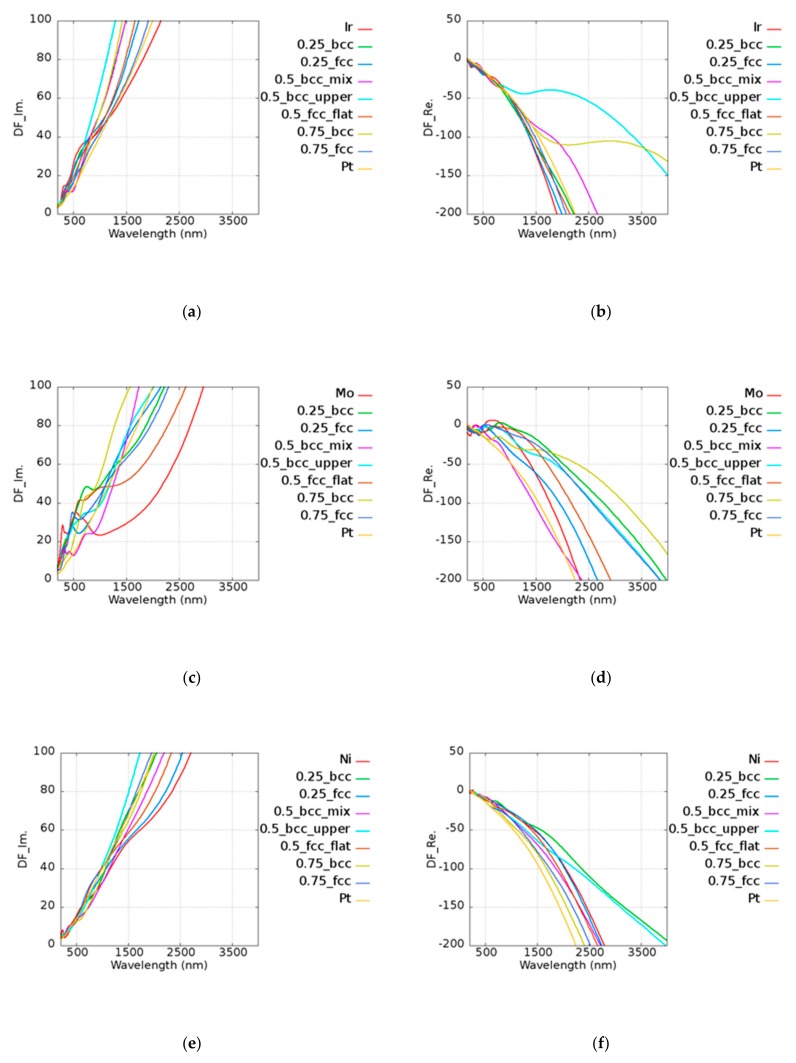

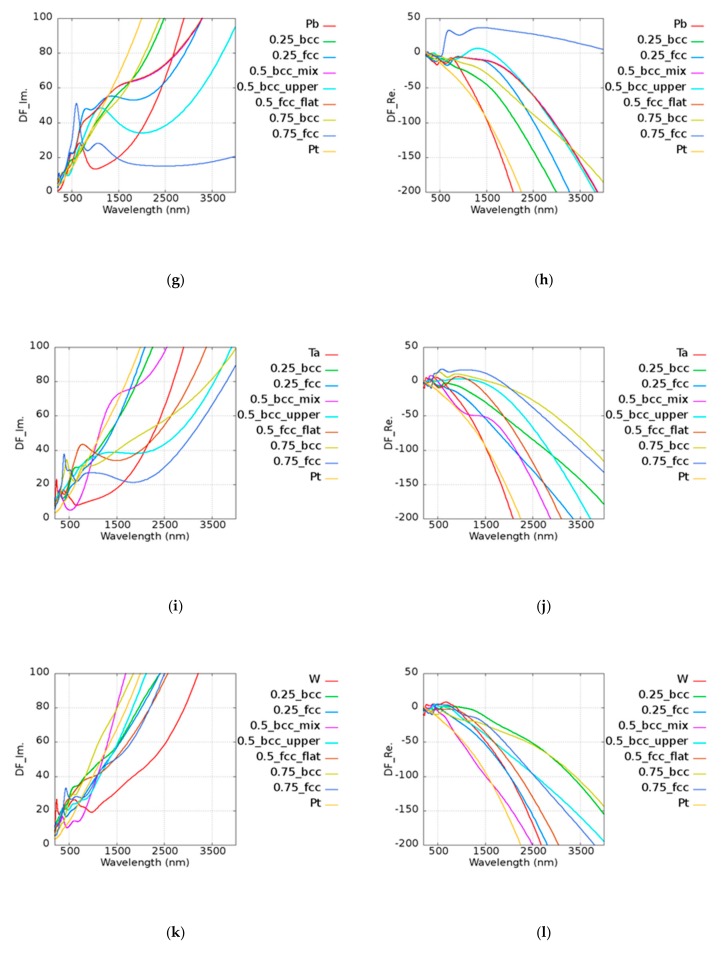
Imaginary and real parts of the permittivity of (**a**,**b**) PtIr, (**c**,**d**) PtMo, (**e**,**f**) PtNi, (**g**,**h**) PtPb, (**i**,**j**) PtTa, and (**k**,**l**) PtW. DF_Im. and DF_Re. along the *y*-axes denote the imaginary and real parts of the dielectric functions.

Unlike pure Au, Pt has a smaller *Q* due to its high imaginary part of the permittivity. The results of *Q* of Pt-based alloy are shown in Figure 7. In the legend of the plots, the first and the last ones characterize the pure metallic elements. The number represents the atomic ratio of Au, while the text after the number denotes the structure and the morphology under the given atomic ratio condition. For example, in Figure 7a, 0.75_fcc represents Au_0.75_Ir_0.25_ with an FCC structure. Pt_0.5_Ir_0.5_ (BCC_mix) and Pt_0.75_Ir_0.25_ (BCC) had stronger plasmonic responses in the visible range of 600 to 700 nm as compared to pure Pt. Pt_0.75_Mo_0.25_ (BCC_mix) exhibited a higher *Q* in the range of 900 to 1300 nm, which contributes to the lower loss and similar real part of pure Pt. The real and imaginary parts of permittivities of Pt-based alloy are shown in Figure A2 of Appendix A. Pt_0.75_Ni_0.25_ (BCC) could be applied in the range of 600 to 1000 nm and Pt_0.25_Ni_0.75_ (FCC) had a similar *Q* to that of two pure metals in the range of 2400 to 2700 nm. Pt_0.25_Pb_0.75_ (FCC) and Pt_0.5_Pb_0.5_ (BCC_upper) exhibited better results above 2750 nm compared to pure Pt. Pt_0.5_Ta_0.5_ (BCC_mix) was good at visible range, and Pt_0.5_Ta_0.5_ (BCC_upper) and Pt_0.5_Ta_0.5_ (BCC_flat) could be applied above 2700 and 2000 nm, respectively. Pt_0.5_Ta_0.5_ (BCC_mix) had a high *Q* in the broad band of 600 to 1400 nm. Pt was found to be a good candidate for catalysis, as mentioned in the previous discussions. It was shown that some alloys possess better performances than pure Pt. According to our simulation results, the design of the material used in devices may be adjusted in different applications in consideration of the cost and the performance.

## 6. Discussions

Most of the cases exhibited fine performances as *Q* > 1 in the infrared frequency, which indicated the good applicability of most alloys. It was found that *Q* was approximated to be inversely proportional to the frequency in the infrared frequency region because it is dominated by the motion of free electron according to the Drude model, as the incident photon energy is low in the infrared region.

For the applications of magnetic plasmonics, the ferromagnetic metal Ni was alloyed with Au and Pt. Our calculated optical property of Au_0.75_Ni_0.25_ has the similar trend as compared to the experimental data by using dual magnetron sputtering to deposit the metallic alloy thin film from Mcpherson et al. [44]. Lonergan et al. [45] studied the effect of different ratios of Pt_x_Ni_(1−x)_ on benzene hydrogenation, and it was shown that Ni in alloy strengthens the bond formation which increases the activity of catalysts. The magnetic moments of Au_x_Ni_(1−x)_ and Pt_x_Ni_(1−x)_ alloy are listed in Table 3. As the content of Ni increases, the magnetic moment rises. However, a trade-off between plasmonic properties and magnetic properties was observed, except that FCC Au_0.5_Ni_0.5_ exhibited better characteristics than pure Ni around 3000 to 4000 nm, which may indicate its potential as a magnetic plasmonic material.

It has been reported that PtMo is suitable for use in fuel cells due to its greater stability compared to that of pure Pt. Pt cathodes suffer from oxidation and a loss of active surface area. It was shown that the alloy cathode had a lighter material loss when CO was present in the fuel stream. The PtMo alloy nanoparticles were reported by Liu [46], who successfully mitigated the contamination of the anode electrocatalysts. Ehteshami et al. [47] also showed the identical trend that PtMo had a better performance than pure Pt. It is worth mentioning that PtNi has also been reported in the literature. It was shown that the bimetallic alloy is more stable than a monometallic material in some cases, making it more suitable for catalytic and thermal applications.

## 7. Optical Responses of Nanorods

To further investigate the optical responses of some practical nano objects, the electromagnetic fields of nanorods made of different materials were examined. In this part, we mainly focused on the effect of the material; therefore, the nanorods were simulated. The influence of the gaps between adjacent nanoraods or the density of nanorods was neglected.

**Figure 8:**
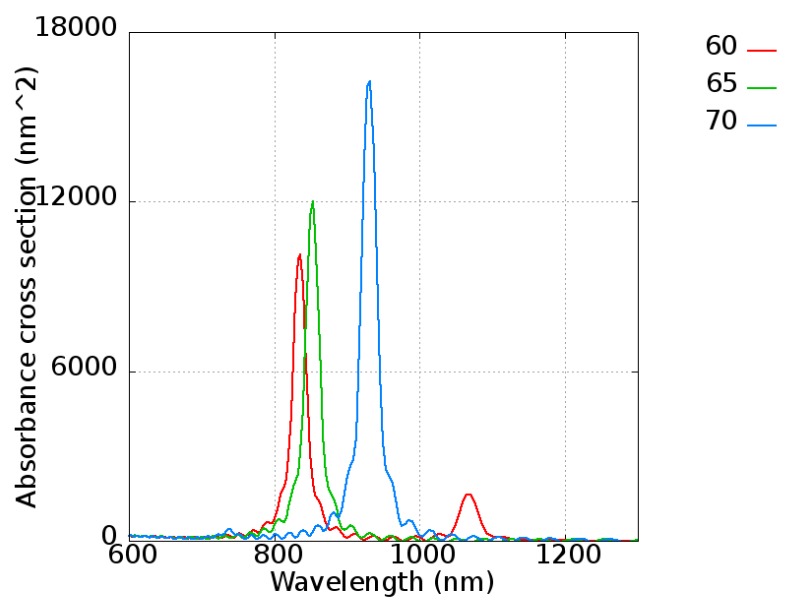
Absorbance cross-sections of the gold nanorods with different nanorod lengths of 60, 65, and 70 nm.

First, to confirm the results of FDTD with the experimental data from Takahashi et al. [48], a gold nanorod with a length varying from 60 to 70 nm with an interval of 5 nm and a radius of 11 nm was simulated. The absorbance of gold nanorods is shown in Figure 8, revealing peaks located at 830, 850, and 950 nm for nanorod lengths of 60, 65, and 70 nm, respectively. Because a single gold nanorod was considered in this simulation, the extreme narrow peak is shown, which is the ideal case of the pure gold nanorod. It is easy to explain that the broadened peak of the experimental data [48] is simply due to the size distribution of the nanorods. On the other hand, the wavelength tunability is clearly shown, indicating that the absorbance peak increased as the nanorod length increased. The intensity was also strengthened with length, which give a quite intuitive explanation of absorbing more energy. The results were consistent with the experimental data [48], showing an absorbance peak located at 800 to 1100 nm.

**Figure 9:**
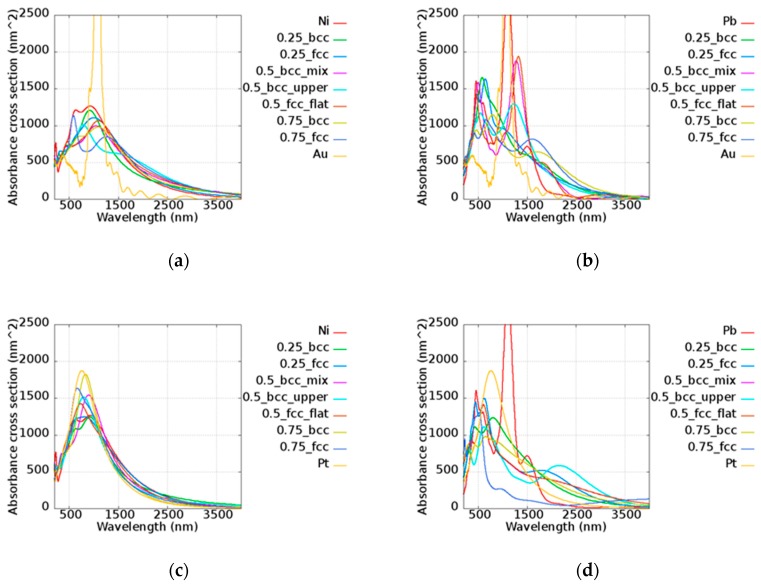
Absorbance cross-sections of the (**a**) AuNi, (**b**) AuPb, (**c**) PtNi, and (**d**) PtPb nanorods with a length of 100 nm and a radius of 11 nm.

The simulated results of the alloy nanorods with a length of 100 nm and a diameter of 11 nm which have cross-section areas of approximately 1480 nm^2^ are shown in Figure 9, including AuNi, AuPb, PtNi, and PtPb. Combining the two materials, the trend of the absorbance cross-section was consistent with the *Q* values. For example, in the AuPb case, the sequence of *Q* intensity above 1000 nm was Au, Pb, Au_0.5_Pb_0.5_ (FCC_flat), and Au_0.5_Pb_0.5_(BCC_mix), etc. However, the nanorod peak absorbance was confined to a small region due to the structure effect of the nanorods. The extreme narrow peak of Au is shown, illustrating its high sensitivity. On the other hand, Pt had a strong absorbance in the visible and infrared light regions, Ni showed quite a broad band from 500 to 1300 nm, and Pb possessed two main peaks at around 490 and 1100 nm. The broad band absorbance of Ni and Pb contributed to the additional absorption in the alloy above 2000 nm compared to the pure metal, which may be applied in the lower energy region. In Au-based alloy nanorods, the better plasmonic performance of alloy was demonstrated compared to pure Au in the visible region. The other better performance was shown in the infrared region around 1300 to 1400 nm in all cases compared to Au. It is worth mentioning that the relative narrow bands of Au_0.5_Pb_0.5_ (FCC_flat), Au_0.5_Pb_0.5_ (FCC_upper), and Au_0.5_Pb_0.5_ (BCC_mix) were found and that all peaks were situated in a similar frequency This could reduce the difficulty in synthesis by allowing some ambiguity in stoichiometry. Peak shifting was shown in most cases except for PtIr and PtNi. Due to the analogous electromagnetic response of Pt, Ir, and Ni, the alloys of these metals maintain similar operation wavelengths of 700 to 1100 nm. These alloys can be well designed by tuning their structure parameters for suitable frequency. On the contrary, the wavelength selective absorbance is shown, which may be utilized for different applications in different operation frequency ranges.

**Figure 10:**
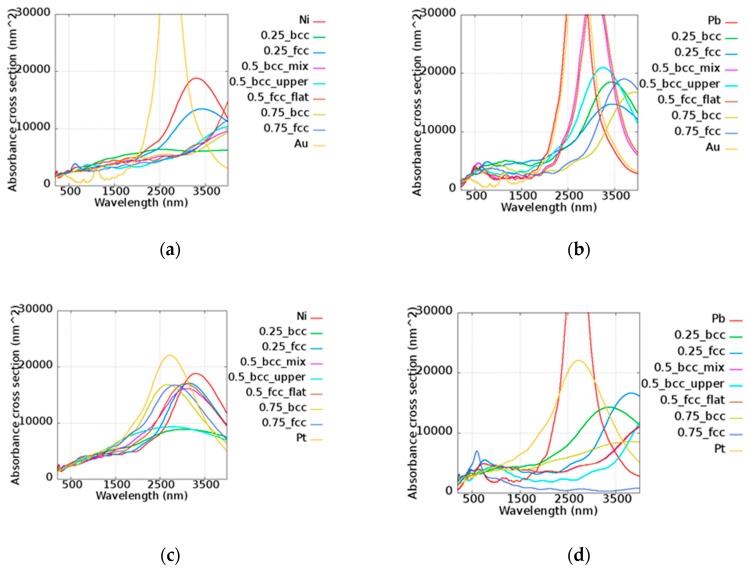
Absorbance cross-sections of the (**a**) AuNi, (**b**) AuPb, (**c**) PtNi, and (**d**) PtPb nanorods with a length of 350 nm and a radius of 11 nm.

The tunable absorbances of nanorods were consisted with the aspect ratio, as demonstrated in many reports [49,50,51]. The nanorod was lengthened to be 350 nm whereas the diameter was maintained at 11 nm. Upon increasing the aspect ratio to the value of 31.81, the absorbance cross-sections of AuNi, AuPb, PtNi, and PtPb varied, as shown in Figure 10. The absorbance peak shifted to around 3000 nm. The absorbance cross-sections of all alloy nanorods possessed a much larger area than the area cross-sections of about 5329.34 nm^2^ around the wavelength of 3000 nm. This indicates that these alloy nanorods are suitable for application in the infrared range. Effects of the wavelength tunability and associated amplification were found in different alloys. This illustrates that the compositions of two elements can be well adjusted to access different absorbance features.

## 8. Conclusions

In this work, the dielectric functions of Au- and Pt-based alloys were examined by DFT simulation, and then adopted in the absorbance calculations of alloy nanorods by FDTD simulations. The plasmonic performances of bulk devices were inspected by their quality factors. Most of the alloys were found to be suitable for use in the infrared region for certain compositions and would be useful for thermal emitters and infrared sensors which can be further employed in MEMS-based systems and micromachines. Furthermore, some of the alloy exhibited better performances in the infrared region compared to pure elemental metals, which is rarely seen for cases in the visible region. Pt-based alloys possessed a similar quality factor *Q* as that of pure metal in the infrared region, which may indicate their potential for replacing Pt in some catalytic applications. FDTD simulations for nanorods were demonstrated to examine the optical responses of these alloys. Wavelength selective properties were shown in most cases from the visible to the infrared region. The nanorods of different alloys could be applied in high-temperature environments, localized surface plasmon polaritions, and catalysts, with considering their *Q* performances at particular wavelengths and manufacturing cost. This work provides a method for designing suitable alloys for infrared devices.

## Figures and Tables

**Table 1:** Space group of the elements Au, Pt, Ni, Ir, Pb, Mo, Ta, and W.

Space Group	F3m3	I3m3
Composition	Au, Pt, Ir, Ni, Pb	Mo, Ta, W

**Table 2:** Different damping frequency of gold reported by Blaber [40], Ordal [41], and Zeman [42].

Damping Frequency (eV)	Blaber [40]	Ordal [41]	Zeman [42]
Au	0.0184	0.0267	0.0708

**Table 3:** Magnetic moments of AuNi and PtNi with different ratios and morphologies.

Magnetic Moment	0	0.25	0.5	0.75	1
Au_x_Ni_(1−x)_	0.617	1.573 (BCC) 1.551 (FCC)	0.496 (BCC_mix) 0.960 (BCC_upper) 0.726 (FCC)	0.006 (BCC) 0.012 (FCC)	0
Pt_x_Ni_(1−x)_	0.617	2.240 (BCC) 2.246 (FCC)	0.705 (BCC_mix) 1.370 (BCC_upper) 2.179 (FCC)	−0.001 (BCC) 1.043 (FCC)	0

## Appendix A

The permittivity of the Au-based alloy and that of the Pt-based alloy are shown in Figure A1 and Figure A2, respectively. The left-hand column is the imaginary part of the permittivity and the right-hand column is real part of the permittivity.

## References

[1] Dao T.D., Chen K., Ishii S., Ohi A., Nabatame T., Kitajima M., Nagao T. (2015). Infrared perfect absorbers fabricated by colloidal mask etching of Al–Al_2_O_3_–Al trilayers. ACS Photonics.

[2] Yang Z.-Y., Ishii S., Yokoyama T., Dao T.D., Sun M.-G., Pankin P.S., Timofeev I.V., Nagao T., Chen K.-P. (2017). Narrowband wavelength selective thermal emitters by confined tamm plasmon polaritons. ACS Photonics.

[3] Yokoyama T., Dao T.D., Chen K., Ishii S., Sugavaneshwar R.P., Kitajima M., Nagao T. (2016). Spectrally selective mid-infrared thermal emission from molybdenum plasmonic metamaterial operated up to 1000 C. Adv. Opt. Mater..

[4] Reddy H., Guler U., Kudyshev Z., Kildishev A.V., Shalaev V.M., Boltasseva A. (2017). Temperature-dependent optical properties of plasmonic titanium nitride thin films. ACS Photonics.

[5] Kaur M., Ishii S., Shinde S.L., Nagao T. (2017). All-ceramic microfibrous solar steam generator: TiN plasmonic nanoparticle-loaded transparent microfibers. ACS Sustain. Chem. Eng.

[6] Sugavaneshwar R.P., Ishii S., Dao T.D., Ohi A., Nabatame T., Nagao T. (2017). Fabrication of highly metallic TiN films by pulsed laser deposition method for plasmonic applications. ACS Photonics.

[7] Bansal A., Verma S. (2015). Optical response of noble metal alloy nanostructures. Phys. Lett. A.

[8] Dodson S., Haggui M., Bachelot R., Plain J., Li S., Xiong Q. (2013). Optimizing electromagnetic hotspots in plasmonic bowtie nanoantennae. J. Phys. Chem. Lett..

[9] Brullot W., Valev V.K., Verbiest T. (2012). Magnetic-plasmonic nanoparticles for the life sciences: Calculated optical properties of hybrid structures. Nanomed. Nanotechnol. Biol. Med..

[10] Kwizera E.A., Chaffin E., Shen X., Chen J., Zou Q., Wu Z., Gai Z., Bhana S., O’Connor R., Wang L. (2016). Size-and shape-controlled synthesis and properties of magnetic–plasmonic core–shell nanoparticles. J. Phys. Chem. C.

[11] Liu D., Wang X., He D., Dao T.D., Nagao T., Weng Q., Tang D., Wang X., Tian W., Golberg D. (2014). Magnetically Assembled Ni@Ag Urchin-Like Ensembles with Ultra-Sharp Tips and Numerous Gaps for SERS Applications. Small.

[12] Zhang L., Xu J., Mi L., Gong H., Jiang S., Yu Q. (2012). Multifunctional magnetic–plasmonic nanoparticles for fast concentration and sensitive detection of bacteria using SERS. Biosens. Bioelectron..

[13] Song H., Meng X., Dao T.D., Zhou W., Liu H., Shi L., Zhang H., Nagao T., Kako T., Ye J. (2017). Light-Enhanced Carbon Dioxide Activation and Conversion by Effective Plasmonic Coupling Effect of Pt and Au Nanoparticles. ACS Appl. Mater. Interfaces.

[14] Bora T., Zoepfl D., Dutta J. (2016). Importance of plasmonic heating on visible light driven photocatalysis of gold nanoparticle decorated zinc oxide nanorods. Sci. Rep..

[15] Mukherjee S., Libisch F., Large N., Neumann O., Brown L.V., Cheng J., Lassiter J.B., Carter E.A., Nordlander P., Halas N.J. (2012). Hot electrons do the impossible: Plasmon-induced dissociation of H2 on Au. Nano Lett..

[16] Xiao T.-H., Cheng Z., Goda K. (2017). Graphene-on-silicon hybrid plasmonic-photonic integrated circuits. Nanotechnology.

[17] Chen C., Wang Z., Wu K., Chong H., Xu Z., Ye H. (2018). ITO–TiN–ITO Sandwiches for Near-Infrared Plasmonic Materials. ACS Appl. Mater. Interfaces.

[18] Franzen S. (2008). Surface plasmon polaritons and screened plasma absorption in indium tin oxide compared to silver and gold. J. Phys. Chem. C.

[19] De Silva K., Gentle A., Arnold M., Keast V., Cortie M. (2015). Dielectric function and its predicted effect on localized plasmon resonances of equiatomic Au–Cu. J. Phys. D Appl. Phys..

[20] Keast V.J., Barnett R.L., Cortie M. (2014). First principles calculations of the optical and plasmonic response of Au alloys and intermetallic compounds. J. Phys. Condens. Matter.

[21] Gong C., Kaplan A., Benson Z.A., Baker D.R., McClure J.P., Rocha A.R., Leite M.S. (2018). Band Structure Engineering by Alloying for Photonics. Adv. Opt. Mater..

[22] Blaber M.G., Arnold M.D., Ford M.J. (2010). A review of the optical properties of alloys and intermetallics for plasmonics. J. Phys. Condens. Matter.

[23] Rakhtsaum G. (2013). Platinum alloys: A selective review of the available literature. Platin. Met. Rev..

[24] McMahon J.M., Schatz G.C., Gray S.K. (2013). Plasmonics in the ultraviolet with the poor metals Al, Ga, In, Sn, Tl, Pb, and Bi. Phys. Chem. Chem. Phys..

[25] Buschow K.V., Van Engen P., Jongebreur R. (1983). Magneto-optical properties of metallic ferromagnetic materials. J. Magn. Magn. Mater..

[26] Leroux C., Cadeville M., Pierron-Bohnes V., Inden G., Hinz F. (1988). Comparative investigation of structural and transport properties of L10 NiPt and CoPt phases; the role of magnetism. J. Phys. F Met. Phys..

[27] Ocken H., Van Vucht J. (1968). Phase equilibria and superconductivity in the molybdenum-platinum system. J. Less Common Met..

[28] Hellenbrandt M. (2004). The inorganic crystal structure database (ICSD)—Present and future. Crystallogr. Rev..

[29] Perdew J.P., Burke K., Ernzerhof M. (1996). Generalized gradient approximation made simple. Phys. Rev. Lett..

[30] Monkhorst H.J., Pack J.D. (1976). Special points for Brillouin-zone integrations. Phys. Rev. B.

[31] Babar S., Weaver J. (2015). Optical constants of Cu, Ag, and Au revisited. Appl. Opt..

[32] Hagemann H.-J., Gudat W., Kunz C. (1975). Optical constants from the far infrared to the x-ray region: Mg, Al, Cu, Ag, Au, Bi, C, and Al_2_O_3_. JOSA.

[33] Johnson P.B., Christy R.-W. (1972). Optical constants of the noble metals. Phys. Rev. B.

[34] McPeak K.M., Jayanti S.V., Kress S.J., Meyer S., Iotti S., Rossinelli A., Norris D.J. (2015). Plasmonic films can easily be better: Rules and recipes. ACS Photonics.

[35] Olmon R.L., Slovick B., Johnson T.W., Shelton D., Oh S.-H., Boreman G.D., Raschke M.B. (2012). Optical dielectric function of gold. Phys. Rev. B.

[36] Philipp H., Palik E.D., Palik E.D. (1985). Handbook of Optical Constants of Solids.

[37] Rioux D., Vallieres S., Besner S., Muñoz P., Mazur E., Meunier M. (2014). An analytic model for the dielectric function of Au, Ag, and their alloys. Adv. Opt. Mater..

[38] Werner W.S., Glantschnig K., Ambrosch-Draxl C. (2009). Optical constants and inelastic electron-scattering data for 17 elemental metals. J. Phys. Chem. Ref. Data.

[39] Windt D.L., Cash W.C., Scott M., Arendt P., Newnam B., Fisher R., Swartzlander A. (1988). Optical constants for thin films of Ti, Zr, Nb, Mo, Ru, Rh, Pd, Ag, Hf, Ta, W, Re, Ir, Os, Pt, and Au from 24 Å to 1216 Å. Appl. Opt..

[40] Blaber M., Arnold M., Ford M. (2010). Designing materials for plasmonic systems: The alkali–noble intermetallics. J. Phys. Condens. Matter.

[41] Ordal M.A., Bell R.J., Alexander R.W., Newquist L.A., Querry M.R. (1988). Optical properties of Al, Fe, Ti, Ta, W, and Mo at submillimeter wavelengths. Appl. Opt..

[42] Zeman E.J., Schatz G.C. (1987). An accurate electromagnetic theory study of surface enhancement factors for silver, gold, copper, lithium, sodium, aluminum, gallium, indium, zinc, and cadmium. J. Phys. Chem..

[43] Jain C., Tuniz A., Reuther K., Wieduwilt T., Rettenmayr M., Schmidt M.A. (2016). Micron-sized gold-nickel alloy wire integrated silica optical fibers. Opt. Mater. Express.

[44] McPherson D.J., Supansomboon S., Zwan B., Keast V.J., Cortie D.L., Gentle A., Dowd A., Cortie M.B. (2014). Strategies to control the spectral properties of Au–Ni thin films. Thin Solid Films.

[45] Lonergan W.W., Vlachos D.G., Chen J.G. (2010). Correlating extent of Pt–Ni bond formation with low-temperature hydrogenation of benzene and 1, 3-butadiene over supported Pt/Ni bimetallic catalysts. J. Catal..

[46] Liu Z., Hu J.E., Wang Q., Gaskell K., Frenkel A.I., Jackson G.S., Eichhorn B. (2009). PtMo Alloy and MoO x@ Pt Core–Shell Nanoparticles as Highly CO-Tolerant Electrocatalysts. J. Am. Chem. Soc..

[47] Ehteshami S.M.M., Jia Q., Halder A., Chan S., Mukerjee S. (2013). The role of electronic properties of Pt and Pt alloys for enhanced reformate electro-oxidation in polymer electrolyte membrane fuel cells. Electrochim. Acta.

[48] Takahashi H., Niidome Y., Niidome T., Kaneko K., Kawasaki H., Yamada S. (2006). Modification of gold nanorods using phosphatidylcholine to reduce cytotoxicity. Langmuir.

[49] Jain P.K., Lee K.S., El-Sayed I.H., El-Sayed M.A. (2006). Calculated absorption and scattering properties of gold nanoparticles of different size, shape, and composition: Applications in biological imaging and biomedicine. J. Phys. Chem. B.

[50] Gao J., Bender C.M., Murphy C.J. (2003). Dependence of the gold nanorod aspect ratio on the nature of the directing surfactant in aqueous solution. Langmuir.

[51] Link S., Mohamed M., El-Sayed M. (1999). Simulation of the optical absorption spectra of gold nanorods as a function of their aspect ratio and the effect of the medium dielectric constant. J. Phys. Chem. B.

